# Effect of an Interdisciplinary CKD Clinic on Disease Progression, Health Care Use, and Social Determinants of Health

**DOI:** 10.34067/KID.0000000734

**Published:** 2025-02-18

**Authors:** Maria B. Mateo Chavez, Andrea Moran-Melendez, Lucy Salter, Lisa Vaughan, Ricardo J. Loor-Torres, Robert Albright, Sandhya Manohar, Ziad Zoghby, Andrea Kattah, Lourdes Gonzalez Suarez, Katie Rose, Vicky Hines, Daniel A. Gonzalez-Mosquera, Satya Sri Bandi, Kasey R. Boehmer

**Affiliations:** 1 Knowledge and Evaluation Research Unit, Division of Endocrinology and Diabetes, Mayo Clinic, Rochester, Minnesota; 2 Department of Psychology, Florida State University, Tallahassee, Florida; 3 Indiana University School of Medicine, Bloomington, Indiana; 4 Department of Quantitative Health Sciences, Mayo Clinic, Rochester, Minnesota; 5 Division of Nephrology and Hypertension, Department of Medicine, Mayo Clinic, Rochester, Minnesota; 6 Department of Medicine, Lincoln Medical Center, Bronx, New York; 7 Division of Healthcare Delivery Research, Mayo Clinic, Rochester, Minnesota

**Keywords:** CKD, clinical nephrology, outcomes, patient-centered care, progression of renal failure, social determinants of health

## Abstract

**Key Points:**

- Patient-centered care in an interdisciplinary CKD clinic addresses complex needs and supports comprehensive CKD management.
- Implementation of an interdisciplinary CKD clinic reduced hospital admissions by 26%, emergency department visits by 30%, and increased nephrology consultations.
- Comprehensive care models with integrated registries enhance tailored interventions, leading to improved CKD management outcomes.

**Background:**

CKD is a significant public health issue globally. Its progressive nature calls for innovative care models to mitigate disease progression and enhance patient outcomes. An interdisciplinary clinic model may offer comprehensive care tailored to the needs of patients with CKD. The aim of this study was to evaluate the effect of an interdisciplinary CKD clinic on disease progression, health care utilization, and social determinants of health (SDOH).

**Methods:**

We conducted a retrospective cohort study at the Mayo Clinic in Rochester, Minnesota. The study included 534 patients enrolled in the CKD clinic between March 5, 2021, and May 31, 2022, excluding those who opted out of research. The intervention involved a clinical registry and an interdisciplinary team delivering evidence-based care pathways, patient education, shared decision making, and care coordination. The primary outcomes assessed were CKD progression and health care utilization, while secondary outcomes examined the effect of SDOH.

**Results:**

At entry, the median age was 73 (interquartile range, 64–79) years, with 60% at stage 4 or lower. Clinic implementation correlated with a 26% decrease in hospital admissions (incidence rate ratio [IRR], 0.74; 95% confidence interval [CI], 0.60 to 0.91; *P* = 0.004) and a 30% reduction in emergency visits (IRR, 0.70; 95% CI, 0.57 to 0.87; *P* = 0.001). Nephrology consultations increased by 46% (IRR, 1.46; 95% CI, 1.34 to 1.60; *P* < 0.001), reflecting enhanced specialized care. Lower exercise frequency and unemployment were linked to increased CKD progression and health care usage.

**Conclusions:**

An interdisciplinary CKD clinic supported by a registry can potentially reduce health care utilization among patients with CKD, with SDOH playing a critical role in disease management.

## Visual Abstract

**Figure:**
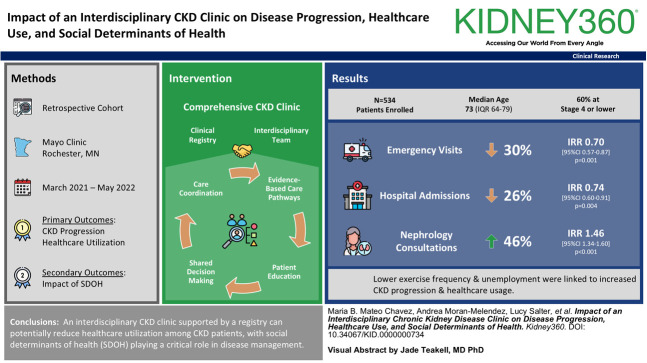

## Introduction

CKD is a growing public health issue in the United States, affecting approximately 37 million people.^1^ It disproportionately affects individuals with hypertension and diabetes, with nearly one-third of diabetics and one-fifth of hypertensive patients at risk.^2^ The often asymptomatic early stages of CKD delay diagnosis and intervention, frequently leading to ESKD.^3,4^ Enhancing early detection and management strategies is essential to improve outcomes in this vulnerable population.

Timely diagnosis and intervention can slow CKD progression, reduce cardiovascular events, hospitalizations, and mortality, thereby easing the burden on health care systems and families. However, both health care providers and patients face barriers to effective ESKD prevention, including limited resources and the complexity of managing multiple comorbidities.^5^ Therefore, a patient-centered approach grounded in self-management is crucial for therapeutic success in CKD.^6^

To address these challenges, researchers have developed electronic tools such as clinical registries to track CKD presentations, treatments, and outcomes, helping tailor interventions to patient needs.^7–9^ These have not only allowed clinicians and researchers to understand patients' needs, but also pave the way for the development of interventions tailored to respond to them.^10^ Using such registries in real-time clinical practice can allow for an interdisciplinary team approach that is tailored to patients' individual needs. For example, when patients reach a certain eGFR, a tailored CKD registry can serve to notify one of the care team members to initiate a discussion about dialysis options.^11^ This approach leverages the entire team and enhances patient understanding of their condition, promotes self-management, and can improve overall health outcomes.^4^

This study evaluates the effect of an interdisciplinary CKD clinic model, including a clinical registry and a team of health care professionals (physicians, nurse practitioners, nurses, dietitians, social workers, and health coaches), on CKD progression, health care utilization, and social determinants of health (SDOH). The clinic model incorporated evidence-based care pathways (*e.g*., recommended vaccinations), patient education (*e.g*., renal dietary information), shared decision making (*e.g*., KRT options), and care coordination support (*e.g*., referral to transplant evaluation) to address the complex needs of patients with CKD.

## Methods

### Study Design and Patient Characteristics

We conducted a retrospective cohort study at Mayo Clinic in Rochester, Minnesota, including adults (18 years or older) under nephrology care, both inpatient and outpatient, who were enrolled in the CKD clinic registry between March 5, 2021, and May 31, 2022. The Mayo Clinic Institutional Review Board approved the study (21-007681). We excluded patients who opted out of chart review under Minnesota Research Authorization law. This report follows the Strengthening the Reporting of Observational Studies in Epidemiology guidelines.^12^

### Interdisciplinary CKD Clinic Intervention Characteristics

The CKD Clinic at Mayo Clinic's Rochester site offers a comprehensive, patient-centered approach for individuals at various CKD stages, aiming to improve quality of life and health outcomes. Patients are typically referred to the CKD clinic by their primary care provider or another specialty provider who identifies the need for nephrology care. After this referral, a nephrologist evaluates whether the patient meets the enrollment criteria for the multidisciplinary CKD clinic, which include residing in the Rochester, Minnesota area or a willingness to travel for care. Enrollment is based solely on patient willingness, with no additional eligibility criteria. Alternatively, patients may choose to continue receiving standard nephrology care without enrolling in the clinic.

Led by nurse practitioners, the clinic team includes nurse educators (navigators), a Capacity Coach,^13^ registered dietitians, and social workers. This holistic care model is supported by a CKD Clinic registry that tracks patients and triggers alerts for follow-up needs, such as when a patient's eGFR falls below 20 ml/min without a dialysis access plan, prompting team interventions.

Care delivery is tailored to CKD progression, with greater support provided as the disease advances. Participants with stages 3, 4, and 5 CKD are primarily managed by advanced practice providers, with nephrologists providing oversight for complex cases. Patients with multiple comorbidities or other complex conditions remain under the direct care of a nephrologist. Although patients with CKD stage 3 and higher are most referred to the clinic, those with stages 1 and 2 can also voluntarily enroll to access additional education and support through the multidisciplinary care (MDC) model.

The clinic offers interdisciplinary appointments, renal function laboratory results, anemia and infectious disease screening, bone metabolism evaluations, immunizations, and referrals to educational programs and transplant services as needed. For early-stage CKD (stages 1 and 2), care focuses on biannual provider visits and renal function tests. For stage 3, the model includes more frequent laboratory results, provider appointments, updated immunizations, patient education, and dietitian visits. For stages 4 and 5, care emphasizes comprehensive coordination, preparation for KRT, enhanced education, and diet management to ensure timely and effective intervention. A detailed description of the clinic's consultation cadence and scheduling intervals is presented in Supplemental Material 1.

### Study Outcomes and Data Collection

The primary outcome was the effect of the interdisciplinary CKD clinic model on CKD progression, assessed by CKD stage and health care utilization rates. Secondary outcomes included associations between SDOH and these clinical outcomes. Baseline was defined as the date of CKD clinic entry. Demographic data, including age, sex, ethnicity, registry entry dates, and insurance status, were collected from the electronic health record (EHR), along with comorbidities such as chronic pain conditions.

SDOH data, extracted from the Mayo Clinic's annual SDOH survey, included variables such as communication frequency with friends or family, access to transportation, employment status, financial difficulties, food security, marital status, and exercise frequency. Health care utilization data included hospital admissions, emergency department (ED) visits, nephrology visits, and primary care visits for 6 months before and after clinic entry. In addition, registry completion metrics—such as educational visits, dietitian and social work consultations, kidney replacement and access plans, home dialysis discussions, transplant referrals, and hepatitis B vaccination completion—were evaluated.

Health care contact days and encounters (*e.g*., ED visits, hospitalizations, and specialty or primary care appointments) were identified from all billed encounters, including in-person, virtual, or home-based visits. This approach ensured comprehensive capture of utilization patterns, accounting for care delivery changes during the coronavirus disease 2019 pandemic. Clinical outcomes of interest included CKD stage progression, as defined by the Kidney Disease Improving Global Outcomes 2024 guidelines.^14^ CKD staging data were automatically updated from the registry on the basis of the latest EHR input.

Data extraction was performed by two trained researchers under the supervision of the lead investigator, with quality checks conducted through duplicate extractions and review by the lead investigator and an experienced statistician to ensure accuracy and reproducibility. Additional information about the study design, outcomes, and data collection methods are described in Supplemental Material 2.

### Statistical Analysis

Summary statistics were reported as median (interquartile range [IQR]) for continuous variables and *n* (%) for categorical variables. Negative binomial regression models were fit to examine health care utilization in the 6-month period before and after CKD clinic entry, and incidence rate ratios (IRRs), along with their corresponding 95% confidence intervals (CIs) and *P* values, were reported. In these models, the duration of follow-up before and after CKD clinic entry served as an offset term, and robust sandwich covariance estimates were employed to account for patients contributing follow-up time to both periods. Models were fitted both unadjusted and adjusted for CKD stage at CKD clinic entry. Additional analyses were also conducted to assess the relationship between SDOH and health care utilization post-CKD clinic entry using negative binomial regression and similar methods as prior models. Proportional odds models were fit to examine the association between worsening CKD stage post-CKD clinic entry and SDOH status, where worsening CKD stage was treated as an ordinal outcome, and the model was adjusted for CKD stage at clinic entry. Odds ratios (ORs), along with their respective 95% CIs and *P* values, were reported for these models, with ORs reflecting the progress to a more severe CKD stage after clinic entry. Statistical significance was defined as two-tailed *P* values < 0.05. All statistical analyses were conducted using SAS 9.4 and R 4.1.3.

## Results

### Cohort Characteristics

A total of 577 patients met the eligibility criteria for this study; 43 were excluded due to opting out of Minnesota Research Authorization, leaving 534 patients included in the final cohort for analysis. The baseline characteristics of the cohort are detailed in Table 1. The median age was 73 years (IQR, 64–79), with the majority being male (54.3%) and White (92.5%). Most patients entered the clinic at CKD stage 4, representing 46.9% of cases, with a median eGFR of 26 ml/min per 1.73 m^2^ (IQR, 19–35).

**Table 1:** Patient characteristics including demographics, clinical, socioeconomic, comorbidities, and outcomes at CKD clinic entry

Patient Characteristic	Total (*N*=534)
**Demographics**	
Age at CKD clinic entry, yr, median (IQR)	73 (64–79)
Biologic sex, *n* (%)	
*Female*	244 (45.7)
*Male*	290 (54.3)
Marital status, *n* (%)	
*Missing*	3
*Divorced*	64 (12.1)
*Living with Partner*	13 (2.4)
*Married*	329 (62.0)
*Single*	51 (9.6)
*Widowed*	74 (13.9)
Ethnicity, *n* (%)	
*Choose not to disclose*	7 (1.3)
*Hispanic/Latino*	21 (3.9)
*Not Hispanic/Latino*	506 (94.8)
Race, *n* (%)	
*American Indian/Alaskan Native*	1 (0.2)
*Asian*	10 (1.9)
*Black*	17 (3.2)
*Choose not to disclose*	4 (0.7)
*Other*	8 (1.5)
*White*	494 (92.5)
**Comorbidities**	
No. of comorbidities, *n* (%)	
*Missing*	2
*0*	4 (0.8)
*1*	3 (0.6)
*2*	6 (1.1)
*3*	23 (4.3)
*4*	29 (5.5)
*5*	55 (10.3)
*6+*	412 (77.4)
Pain condition, *n* (%)	
*Missing*	2
*No*	381 (71.6)
*Yes*	151 (28.4)
**Clinical characteristics**	
Current CKD stage, *n* (%)	
*1*	3 (0.6)
*2*	13 (2.4)
*3a*	46 (8.6)
*3b*	149 (27.9)
*4*	231 (43.3)
*5, not yet dialyzing*	92 (17.2)
CKD stage at CKD clinic entry, *n* (%)	
*Missing*	1
*1*	1 (0.2)
*2*	16 (3.0)
*3a*	50 (9.4)
*3b*	146 (27.4)
*4*	250 (46.9)
*5, not yet dialyzing*	70 (13.1)
Current eGFR, median (IQR)	26.0 (18.0–35.0)
eGFR at CKD clinic entry, median (IQR)	26.0 (19.0–35.0)
**Socioeconomic factors**	
Insured, *n* (%)	
*No*	1 (0.2)
*Yes*	533 (99.8)
Insurance type, *n* (%)	
*Commercial*	79 (14.8)
*Government*	330 (61.8)
*Government and commercial*	123 (23.0)
*International*	1 (0.2)
*None*	1 (0.2)
Communication with friends and family, *n* (%)	
*Missing*	128
*1× weekly*	55 (13.5)
*2× weekly*	33 (8.1)
*3× weekly*	47 (11.6)
*>3× weekly*	251 (61.8)
*Never*	14 (3.4)
*Patient refused*	6 (1.5)
Lack of transportation (medical), *n* (%)	
*Missing*	125
*No*	399 (97.6)
*Yes*	10 (2.4)
Lack of transportation (nonmedical), *n* (%)	
*Missing*	128
*No*	395 (97.3)
*Patient refused*	2 (0.5)
*Yes*	9 (2.2)
Employment, *n* (%)	
*Missing*	19
*Disabled*	44 (8.5)
*Employed*	103 (20.0)
*Other*	4 (0.8)
*Retired*	342 (66.4)
*Unemployed*	22 (4.3)
Difficulty paying living expenses, *n* (%)	
*Missing*	131
*No*	338 (83.9)
*Patient refused*	6 (1.5)
*Yes*	59 (14.6)
Unstable housing last year, *n* (%)	
*Missing*	125
*No*	398 (97.3)
*Patient refused*	5 (1.2)
*Yes*	6 (1.5)
Worried about food last year, *n* (%)	
*Missing*	130
*No*	384 (95.0)
*Patient refused*	5 (1.2)
*Yes*	15 (3.7)
Days exercise per week, *n* (%)	
*Missing*	127
*0 d/wk*	158 (38.8)
*1–4 d/wk*	167 (41.0)
*5+ d/wk*	74 (18.2)
*Patient refused*	8 (2.0)

IQR, interquartile range.

### CKD Progression

#### CKD Clinic Requirements in CKD Stages 4 and 5

**Figure 1:**
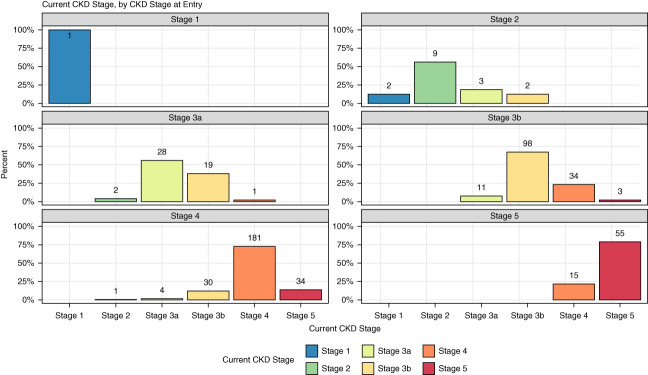
**Bar plot of patient's current CKD stage, stratified by CKD stage at entry.** Paneled bar graphs depict the current CKD stages of patients who entered the interdisciplinary CKD clinic at various CKD stages (1–5). Each panel represents a distinct entry CKD stage, with the corresponding bars illustrating the percentage of patients currently classified into each CKD stage. The *x* axis shows the current CKD stage the patient is in, and the *y* axis denotes the percentage of patients (0%–100%) within each of these stages. The panels collectively demonstrate the progression, stability, or regression of CKD stages over time for patients in the clinic.

Figure 1 shows the progression of CKD stages among patients, with most remaining stable or progressing within their stage. Notably, some patients showed improvement, such as those entering at stage 2, where two improved to stage 1. Conversely, a significant number of patients entering at stages 3b and 4 either progressed to higher stages or remained stable, highlighting the varying trajectories of CKD management.

Among the 158 patients with an eGFR of <20 ml/min per 1.73 m^2^ at the last follow-up, 68 (44.7%) required dietitian support, 81 (53.3%) had participated in education sessions, and 76 (50.0%) had received social work support. A kidney replacement plan was established for 60 patients (39.5%), with in-center dialysis being the most common (33, 21.0%). Notably, 23 patients (15.0%) had an access plan for hemodialysis, and 35 patients (22.9%) were referred for transplant evaluation. Hepatitis B vaccination completion rates were 48.1% for the first dose, 37.3% for the second dose, and 27.8% for the third dose (Table 2).

**Table 2:** CKD clinic requirements among patients with current eGFR <20

CKD Clinic Requirements	Total (*N*=158)
**Dietitian requirement, *n* (%)**	
Missing	6
No	84 (55.3)
Yes	68 (44.7)
**Education requirement, *n* (%)**	
Missing	6
No	71 (46.7)
Yes	81 (53.3)
**Social work requirement, *n* (%)**	
Missing	6
No	76 (50.0)
Yes	76 (50.0)
**Kidney replacement plan, *n* (%)**	
Missing	6
No	92 (60.5)
Yes	60 (39.5)
**Kidney replacement plan type, *n* (%)**	
Missing	1
Home hemodialysis	2 (1.3)
In center dialysis	33 (21.0)
None	94 (59.9)
Opting out of dialysis	8 (5.1)
Other/undecided	2 (1.3)
Peritoneal dialysis	18 (11.5)
**Access plan, *n* (%)**	
Missing	5
No	130 (85.0)
Yes	23 (15.0)
**Access plan type, *n* (%)**	
Missing	1
AV fistula	24 (15.3)
None	133 (84.7)
**Home dialysis options discussed, *n* (%)**	
Missing	5
No	143 (93.5)
Yes	10 (6.5)
**Transplant referral, *n* (%)**	
Missing	5
No	118 (77.1)
Yes	35 (22.9)
**Hep B vaccination Status**	
Hep B first dose, *n* (%)	
*No*	82 (51.9)
*Yes*	76 (48.1)
Hep B second dose, *n* (%)	
*No*	99 (62.7)
*Yes*	59 (37.3)
Hep B third dose, *n* (%)	
*No*	114 (72.2)
*Yes*	44 (27.8)

AV, arteriovenous; Hep B, hepatitis B.

#### Health Care Utilization

In unadjusted models, the rate of general hospital admissions decreased by 26% after CKD clinic entry (IRR, 0.74 [95% CI, 0.60 to 0.91]; *P* = 0.004), and similarly, the rate of ED visits also decreased by 30% compared with pre-CKD clinic entry (IRR, 0.70 [95% CI, 0.57 to 0.87]; *P* = 0.001). Conversely, nephrology visit rates increased by 46% postclinic entry (IRR, 1.46 [95% CI, 1.34 to 1.60]; *P* < 0.001), while primary care visit rates decreased by 22% after clinic entry (IRR, 0.78 [95% CI, 0.69 to 0.88]; *P* < 0.001; Table 3). These findings remained significant in models adjusted for CKD stage at entry. A summary of the patient data and number of encounters of each type before and after CKD clinic entry is provided in Supplemental Table 1.

**Table 3:** Health care utilization after CKD clinic entry

Patient Encounter Type	Unadjusted for CKD Stage at Entry		Adjusted for CKD Stage at Entry	
	Post-CKD Clinic Entry IRR (95% CI)	*P* Value	Post-CKD Clinic Entry IRR (95% CI)	*P* Value
General hospital admissions	0.74 (0.60 to 0.91)	0.004	0.72 (0.58 to 0.88)	0.002
ED visits	0.70 (0.57 to 0.87)	0.001	0.70 (0.57 to 0.86)	<0.001
Nephrology visits (in-center or virtual)	1.46 (1.34 to 1.60)	<0.001	NE	NE
Primary care visits	0.78 (0.69 to 0.88)	<0.001	0.78 (0.69 to 0.88)	<0.001

CI, confidence interval; ED, emergency department; IRR, incidence rate ratio, NE, not estimable due to sparsity of cells.

#### Effect of SDOH

Table 4 presents the associations between SDOH metrics and hospitalizations, ED visit rates, and CKD progression after clinic entry, adjusted for baseline CKD stage. Notably, patients who reported exercising fewer than 5 days per week were associated with a higher risk of CKD progression. Specifically, those who exercised 1–4 days per week had an OR of 2.01 (95% CI, 1.09 to 3.70; *P* = 0.026) for CKD progression compared with those who exercised five or more days per week, whereas patients who did not exercise at all had an OR of 2.21 (95% CI, 1.19 to 4.11; *P* = 0.012). In addition, patients who were disabled or unemployed were associated with a higher odd of CKD progression compared with those who were employed or retired (OR, 1.81 [95% CI, 1.03 to 3.15]; *P* = 0.038).

**Table 4:** Associations between patient encounter rates and social determinants of health status

SDOH Metric	*N*	Number General Hospital Admissions		Number ED Visits		Change in CKD Stage from Entry	
		IRR (95% CI)	*P* Value	IRR (95% CI)	*P* Value	OR (95% CI)^a^	*P* Value
**Insurance type**							
Government	330	0.85 (0.57 to 1.26)	0.41	0.95 (0.59 to 1.53)	0.83	0.97 (0.62 to 1.53)	0.91
Government+commercial	123	Ref	Ref	Ref	Ref	Ref	Ref
**Difficulty paying living expenses**							
Yes	59	1.47 (0.91 to 2.37)	0.11	1.62 (0.92 to 2.85)	0.10	1.58 (0.86 to 2.90)	0.14
No	338	Ref	Ref	Ref	Ref	Ref	Ref
**Days exercise per week**							
0	158	1.25 (0.72 to 2.16)	0.43	1.57 (0.80 to 3.07)	0.19	2.21 (1.19 to 4.11)	0.012
1–4	167	1.20 (0.70 to 2.06)	0.51	1.93 (1.00 to 3.73)	0.049	2.01 (1.09 to 3.70)	0.026
5+	74	Ref	Ref	Ref	Ref	Ref	Ref
**Communication with friends and family**							
<3× weekly	102	0.77 (0.49 to 1.22)	0.27	0.78 (0.46 to 1.31)	0.34	1.00 (0.61 to 1.63)	>0.99
≥3× weekly	298	Ref	Ref	Ref	Ref	Ref	Ref
**Lack of transportation (medical)**							
No	399	Ref	Ref	Ref	Ref	Ref	Ref
Yes	10	0.76 (0.20 to 2.89)	0.69	1.72 (0.48 to 6.16)	0.40	1.08 (0.27 to 4.28)	0.92
**Lack of transportation (nonmedical)**							
No	395	Ref	Ref	Ref	Ref	Ref	Ref
Yes	9	1.13 (0.33 to 3.87)	0.85	2.28 (0.64 to 8.08)	0.20	0.76 (0.18 to 3.27)	0.71
**Employment**							
Disabled/unemployed	66	1.29 (0.80 to 2.08)	0.29	1.65 (0.95 to 2.87)	0.076	1.81 (1.03 to 3.15)	0.038
Employed/Retired	445	Ref	Ref	Ref	Ref	Ref	Ref
**Worried about food last year**							
No	384	Ref	Ref	Ref	Ref	Ref	Ref
Yes	15	1.11 (0.43 to 2.88)	0.83	1.80 (0.64 to 5.10)	0.27	1.34 (0.43 to 4.22)	0.61

All models adjusted for CKD stage at entry. CI, confidence interval; ED, emergency department; IRR, incidence rate ratio; NE, not estimable due to sparsity of cells; OR, odds ratio; SDOH, social determinants of health.

a OR is modeling the probability of moving to a more severe CKD stage after entry.

## Discussion

This research aimed to evaluate the effect of implementing an interdisciplinary CKD clinic among patients receiving CKD care. Distinct patterns were identified in the distribution of CKD stages on the basis of the initial CKD stage at clinic entry. Notably, patients in the early stages of CKD mostly exhibited either stability or regression, while patients in advanced CKD stages at clinic entry tended to progress to a more severe disease course. In this population, proactive management with the help of the registry is critical to prevent adverse outcomes. Furthermore, our results suggest potential associations between health care utilization and CKD stage progression with exercise frequency and employment status.

Prior literature has linked interdisciplinary clinics with reduced all-cause mortality, lower risk of initiating dialysis, and decreased urgent or inpatient dialysis initiation with a hemodialysis catheter in cohort studies involving patients with late-stage CKD.^15–17^ The incorporation of enhanced nurse care in managing these patients has also demonstrated positive outcomes compared with standard care. A multisite randomized controlled trial reported decreased hospitalizations among patients with late-stage CKD in clinics with increased nurse care manager coordination, improved CKD education, and better preparation for ESKD.^18^ These findings align with the present study, as patients experienced lower rates of hospitalizations, ED visits, and primary care visits after enrollment in the CKD clinic cohort compared with before enrollment.

In this study, the median age of patients was 73 years, with the most common CKD stage at clinic entry being stage 3a. A single-center study conducted on patients with an average age of 60 years and a higher prevalence of CKD stages 4–5 suggested that a team-based approach to CKD care may better support sicker patients with more advanced CKD, enabling them to achieve comparable patient-centered outcomes compared with those with less advanced CKD.^19^ These findings, in relation to this research, highlight the need to further explore which populations would benefit most from these intensive team-based interventions.

SDOH may also influence CKD progression. Associations have been identified between lower socioeconomic status and CKD progression to ESKD, inadequate dialysis treatment, reduced access to kidney transplantation, and poor overall health outcomes.^20^ Racial and ethnic disparities, often linked to lower socioeconomic status in the United States, have also been linked to adverse health outcomes.^21,22^ However, the observed associations in our study between SDOH variables and outcomes may be partly influenced by reverse causality; patients with more advanced CKD could experience greater socioeconomic challenges or reduced physical activity due to their health status. These findings suggest the importance of improving access to comprehensive renal care, which may be limited in some populations. CKD registries could help by identifying high-risk patients earlier in the disease trajectory, supporting timely interventions and recommendations to prevent disease progression, even in settings where frequent in-person visits with a nephrologist are less accessible.

This research introduces a new perspective by illustrating the specific effect of physical activity—a modifiable lifestyle factor—on CKD progression and health care utilization. Our findings indicate an inverse association between the number of days exercising in a week and health care utilization, particularly the number of ED visits during the study period. However, existing literature regarding the effects of physical activity on CKD progression parameters is varied. A randomized controlled trial investigating the effect of lifestyle intervention (*i.e*., access to MDC through a nurse practitioner–led CKD clinic, exercise training, and a lifestyle program) versus usual care showed significant improvement in metabolic equivalent tasks and 6-min walking distance, but these did not translate to improved renal function.^23^ Further research could focus on this understudied area, specifically examining the effects of physical activity on renal function changes in this population.

In our experience, the implementation of a CKD registry has improved our ability to manage nephrology care. Key benefits include its user-intuitive design, which improved tracking of patient appointments and follow-ups, ensuring timely interventions; facilitated communication with patients through a mass mailing service for appointment reminders, educational materials, and health alerts; streamlined monitoring of laboratory results, enabling prompt adjustments to treatment plans; simplified the process of ordering and tracking laboratory tests, reducing administrative burdens; and supported virtual consultations, which were particularly useful during the coronavirus disease 2019 pandemic to ensure continuity of care.

Although the increase in nephrology provider visits was observed, it is essential to evaluate whether this trend is beneficial or burdensome. Increased visits may indicate proactive management and closer monitoring of patients, potentially leading to better outcomes. However, it could also contribute to provider burnout and higher health care costs. To mitigate these issues, we propose leveraging the CKD registry to identify stable patients who may require fewer in-person visits, thus optimizing resource allocation and reducing the burden on nephrology providers.

To enhance the effectiveness of an interdisciplinary CKD clinic, we recommend a comprehensive approach involving a well-coordinated team of health care professionals. Key roles for team members include nephrologists, who lead the medical management of patients with CKD and oversee treatment plans; nurse care managers, who provide patient education, coordinate care, monitor patient progress, and facilitate communication between patients and the health care team; dietitians, who offer nutritional counseling and develop individualized dietary plans to support kidney health and manage comorbidities; social workers, who address SDOH, provide resources for financial assistance, and support patients in accessing community services; pharmacists, who review and manage medications, ensure adherence to prescribed treatments, and educate patients about potential drug interactions; and physical therapists, who design and implement exercise programs tailored to individual patient needs to promote physical activity and overall health.

This study highlights the potential benefits of an interdisciplinary CKD clinic in managing kidney disease progression, particularly emphasizing the role of physical activity and tailored interventions in improving health care utilization. Although our findings indicate reductions in hospitalizations and ED visits among clinic participants, they also suggest that the extent of these benefits may depend on the patient's baseline risk profile. High-risk patients tended to show disease progression despite the interventions, whereas low-risk patients seemed to benefit more from the clinic's proactive approach, which includes ongoing renal function monitoring, patient education, and individualized care plans. These results highlight the importance of tailoring care models to patient needs and continually evaluating their effectiveness.

However, the variability in outcomes observed with MDC models across studies presents challenges in translating process improvements into meaningful clinical outcomes. A cluster randomized trial of a multifaceted intervention, including EHR-based population health management and pharmacist-led medication reviews, improved angiotensin-converting enzyme inhibitor/angiotensin II receptor blockers use, but did not significantly affect CKD progression.^24^ Similarly, a pragmatic trial using an EHR algorithm with practice facilitators showed no reduction in hospitalizations or mortality, despite better delivery of guideline-directed therapies.^25^ These mixed results may reflect limitations such as short follow-up periods, high baseline care quality, and inconsistencies in implementation, all of which can dilute the observed effect of MDC. Future research should address these gaps by extending follow-up, standardizing intervention delivery, and incorporating strategies to improve patient engagement and address broader systemic barriers to care.

Continued research is essential to confirm these associations, explore their mechanisms, and understand the clinic's role in addressing varying patient needs. By filling these gaps, we can enhance our comprehension of CKD progression and develop more effective, personalized management strategies, ultimately improving patient outcomes and reducing the burden of CKD on the health care system.

This study has several limitations, largely stemming from its retrospective design. The absence of a control group and randomization restricts our ability to establish causality. Although hospitalization and ED visit rates showed improvement, unmeasured external factors and natural tendencies, such as regression to the mean, may have influenced these findings. In addition, limitations in the registry design affected our analysis, as it lacked data on the use or titration of kidney-protective therapies (*e.g*., sodium-glucose cotransporter 2 inhibitors and renin-angiotensin-aldosterone system blockade), nephrology-specific outcomes (*e.g*., access-related infection rates), and dialysis initiation methods (*e.g*., planned versus crash-start with temporary dialysis catheters).

Selection bias and survivor selection bias (immortal time bias) are both considerations in this study. Selection bias may have occurred because of voluntary enrollment in the clinic of patients with less complex CKD, potentially overestimating the clinic's effectiveness. Survivor selection bias may have occurred due to systematically differences in patient who completed the follow-up period from those who did not, potentially skewing the results.

The study's single-center setting further limits generalizability, and cross-site comparisons are needed to better understand how differences in patient populations and health care practices influence outcomes. Moreover, the low representation of low-resource groups—such as those facing challenges with transportation, living expenses, or food security—may affect the applicability of findings.

Finally, individual kidney failure risk for each of the clinic participants was not standardized or protocolized within the multidisciplinary clinic model.

Our findings suggest that an interdisciplinary CKD clinic supported by a CKD registry may benefit CKD management. Postclinic entry, patients experienced fewer hospitalizations, ED visits, and primary care visits compared with preclinic entry. Exercise frequency and employment status were linked to reduced health care utilization and slower disease progression. Further research, including randomized trials in diverse populations, is needed to validate this care model.

## Supplementary Material

SUPPLEMENTARY MATERIAL

## Data Availability

Partial restrictions to the data and/or materials apply. The datasets generated during and/or analyzed during the current study are available from the corresponding author on reasonable request.

## Supplemental Material

Supplemental Table 1. Summary of patient encounter rates.

Supplemental Material 1. CKD clinic cadence.

Supplemental Material 2. Protocol.
