# Supplementary material for: Effect of an Interdisciplinary CKD Clinic on Disease Progression, Health Care Use, and Social Determinants of Health
Source: Kidney360. 2025 Feb 18;6(6):937–46. doi: 10.34067/KID.0000000734 (PMC12233851; doi:10.34067/KID.0000000734)
Supplement: SUPPLEMENTARY MATERIAL [file kidney360-6-0937-s001.pdf]

ASN American Society of Nephrology
JASN Journal of the American Society of Nephrology
KIDNEY360 Accessing Our World From Every Angle
CJASN Clinical Journal of the American Society of Nephrology

ASN Journal Disclosure Form

As per ASN journal policy, I have disclosed any financial relationships or commitments I have held in the past 36 months as included below. I have listed my Current Employer below to indicate there is a relationship requiring disclosure. If no relationship exists, my Current Employer is not listed.

R. Albright reports the following:
Employer: Mayo Clinic

I understand that the information above will be published within the journal article, if accepted, and that failure to comply and/or to accurately and completely report the potential financial conflicts of interest could lead to the following: 1) Prior to publication, article rejection, or 2) Post-publication, sanctions ranging from, but not limited to, issuing a correction, reporting the inaccurate information to the authors' institution, banning authors from submitting work to ASN journals for varying lengths of time, and/or retraction of the published work.

Name: Robert C. Albright
Manuscript ID: K360-2024-000797R1
Manuscript Title: Impact of an Interdisciplinary Chronic Kidney Disease Clinic on Disease Progression, Healthcare Use, and Social Determinants of Health
Date of Completion: January 23, 2025
Disclosure Updated Date: January 23, 2025

ASN American Society of Nephrology | JASN Journal of the American Society of Nephrology | KIDNEY360 Accessing Our World From Every Angle | CJASN Clinical Journal of the American Society of Nephrology

ASN Journal Disclosure Form

As per ASN journal policy, I have disclosed any financial relationships or commitments I have held in the past 36 months as included below. I have listed my Current Employer below to indicate there is a relationship requiring disclosure. If no relationship exists, my Current Employer is not listed.

S. Bandi has nothing to disclose.

I understand that the information above will be published within the journal article, if accepted, and that failure to comply and/or to accurately and completely report the potential financial conflicts of interest could lead to the following: 1) Prior to publication, article rejection, or 2) Post-publication, sanctions ranging from, but not limited to, issuing a correction, reporting the inaccurate information to the authors' institution, banning authors from submitting work to ASN journals for varying lengths of time, and/or retraction of the published work.

Name: Satya Sai Sri Bandi
Manuscript ID: K360-2024-000797R1
Manuscript Title: Impact of an Interdisciplinary Chronic Kidney Disease Clinic on Disease Progression, Healthcare Use, and Social Determinants of Health
Date of Completion: December 16, 2024
Disclosure Updated Date: December 16, 2024

ASN American Society of Nephrology | JASN Journal of the American Society of Nephrology | KIDNEY360 Accessing Our World From Every Angle | CJASN Clinical Journal of the American Society of Nephrology

ASN Journal Disclosure Form

As per ASN journal policy, I have disclosed any financial relationships or commitments I have held in the past 36 months as included below. I have listed my Current Employer below to indicate there is a relationship requiring disclosure. If no relationship exists, my Current Employer is not listed.

K. Boehmer has nothing to disclose.

I understand that the information above will be published within the journal article, if accepted, and that failure to comply and/or to accurately and completely report the potential financial conflicts of interest could lead to the following: 1) Prior to publication, article rejection, or 2) Post-publication, sanctions ranging from, but not limited to, issuing a correction, reporting the inaccurate information to the authors' institution, banning authors from submitting work to ASN journals for varying lengths of time, and/or retraction of the published work.

Name: Kasey Boehmer
Manuscript ID: K360-2024-000797R1
Manuscript Title: Impact of an Interdisciplinary Chronic Kidney Disease Clinic on Disease Progression, Healthcare Use, and Social Determinants of Health
Date of Completion: January 28, 2025
Disclosure Updated Date: January 28, 2025

ASN American Society of Nephrology
JASN Journal of the American Society of Nephrology
KIDNEY360 Accessing Our World From Every Angle
CJASN Clinical Journal of the American Society of Nephrology

ASN Journal Disclosure Form

As per ASN journal policy, I have disclosed any financial relationships or commitments I have held in the past 36 months as included below. I have listed my Current Employer below to indicate there is a relationship requiring disclosure. If no relationship exists, my Current Employer is not listed.

D. Gonzalez Mosquera has nothing to disclose.

I understand that the information above will be published within the journal article, if accepted, and that failure to comply and/or to accurately and completely report the potential financial conflicts of interest could lead to the following: 1) Prior to publication, article rejection, or 2) Post-publication, sanctions ranging from, but not limited to, issuing a correction, reporting the inaccurate information to the authors' institution, banning authors from submitting work to ASN journals for varying lengths of time, and/or retraction of the published work.

Name: Daniel A Gonzalez Mosquera
Manuscript ID: K360-2024-000797R1
Manuscript Title: Impact of an Interdisciplinary Chronic Kidney Disease Clinic on Disease Progression, Healthcare Use, and Social Determinants of Health
Date of Completion: December 15, 2024
Disclosure Updated Date: December 15, 2024

ASN Journal Disclosure Form

As per ASN journal policy, I have disclosed any financial relationships or commitments I have held in the past 36 months as included below. I have listed my Current Employer below to indicate there is a relationship requiring disclosure. If no relationship exists, my Current Employer is not listed.

M. Gonzalez Suarez reports the following:
Employer: Mayo Clinic; and Research Funding: AstraZeneca;.

I understand that the information above will be published within the journal article, if accepted, and that failure to comply and/or to accurately and completely report the potential financial conflicts of interest could lead to the following: 1) Prior to publication, article rejection, or 2) Post-publication, sanctions ranging from, but not limited to, issuing a correction, reporting the inaccurate information to the authors' institution, banning authors from submitting work to ASN journals for varying lengths of time, and/or retraction of the published work.

Name: Maria Lourdes Gonzalez Suarez
Manuscript ID: K360-2024-000797R1
Manuscript Title: "Impact of an Interdisciplinary Chronic Kidney Disease Clinic on Disease Progression, Healthcare Use, and Social Determinants of Health
Date of Completion: January 30, 2025
Disclosure Updated Date: January 23, 2025

ASN American Society of Nephrology · JASN Journal of the American Society of Nephrology · KIDNEY360 Accessing Our World From Every Angle · CJASN Clinical Journal of the American Society of Nephrology

ASN Journal Disclosure Form

As per ASN journal policy, I have disclosed any financial relationships or commitments I have held in the past 36 months as included below. I have listed my Current Employer below to indicate there is a relationship requiring disclosure. If no relationship exists, my Current Employer is not listed.

V. Hines has nothing to disclose.

I understand that the information above will be published within the journal article, if accepted, and that failure to comply and/or to accurately and completely report the potential financial conflicts of interest could lead to the following: 1) Prior to publication, article rejection, or 2) Post-publication, sanctions ranging from, but not limited to, issuing a correction, reporting the inaccurate information to the authors' institution, banning authors from submitting work to ASN journals for varying lengths of time, and/or retraction of the published work.

Name: Vicky L. Hines
Manuscript ID: K360-2024-000797R1
Manuscript Title: Impact of an Interdisciplinary Chronic Kidney Disease Clinic on Disease Progression, Healthcare Use, and Social Determinants of Health
Date of Completion: January 24, 2025
Disclosure Updated Date: January 24, 2025

ASN American Society of Nephrology | JASN Journal of the American Society of Nephrology | KIDNEY360 Accessing Our World From Every Angle | CJASN Clinical Journal of the American Society of Nephrology

ASN Journal Disclosure Form

As per ASN journal policy, I have disclosed any financial relationships or commitments I have held in the past 36 months as included below. I have listed my Current Employer below to indicate there is a relationship requiring disclosure. If no relationship exists, my Current Employer is not listed.

A. Kattah reports the following:
Research Funding: Quanta

I understand that the information above will be published within the journal article, if accepted, and that failure to comply and/or to accurately and completely report the potential financial conflicts of interest could lead to the following: 1) Prior to publication, article rejection, or 2) Post-publication, sanctions ranging from, but not limited to, issuing a correction, reporting the inaccurate information to the authors' institution, banning authors from submitting work to ASN journals for varying lengths of time, and/or retraction of the published work.

Name: Andrea G. Kattah
Manuscript ID: K360-2024-000797R1
Manuscript Title: Impact of an Interdisciplinary Chronic Kidney Disease Clinic on Disease Progression, Healthcare Use, and Social Determinants of Health
Date of Completion: January 24, 2025
Disclosure Updated Date: May 30, 2024

ASN American Society of Nephrology | JASN Journal of the American Society of Nephrology | KIDNEY360 Accessing Our World From Every Angle | CJASN Clinical Journal of the American Society of Nephrology

ASN Journal Disclosure Form

As per ASN journal policy, I have disclosed any financial relationships or commitments I have held in the past 36 months as included below. I have listed my Current Employer below to indicate there is a relationship requiring disclosure. If no relationship exists, my Current Employer is not listed.

R. Loor Torres reports the following:
Employer: Mayo Clinic; Ascension Saint Joseph

I understand that the information above will be published within the journal article, if accepted, and that failure to comply and/or to accurately and completely report the potential financial conflicts of interest could lead to the following: 1) Prior to publication, article rejection, or 2) Post-publication, sanctions ranging from, but not limited to, issuing a correction, reporting the inaccurate information to the authors' institution, banning authors from submitting work to ASN journals for varying lengths of time, and/or retraction of the published work.

Name: Ricardo Jose Loor Torres
Manuscript ID: K360-2024-000797R1
Manuscript Title: Impact of an Interdisciplinary Chronic Kidney Disease Clinic on Disease Progression, Healthcare Use, and Social Determinants of Health
Date of Completion: January 23, 2025
Disclosure Updated Date: January 23, 2025

ASN Journal Disclosure Form

As per ASN journal policy, I have disclosed any financial relationships or commitments I have held in the past 36 months as included below. I have listed my Current Employer below to indicate there is a relationship requiring disclosure. If no relationship exists, my Current Employer is not listed.

S. Manohar reports the following:
Employer: Mayo Clinic

I understand that the information above will be published within the journal article, if accepted, and that failure to comply and/or to accurately and completely report the potential financial conflicts of interest could lead to the following: 1) Prior to publication, article rejection, or 2) Post-publication, sanctions ranging from, but not limited to, issuing a correction, reporting the inaccurate information to the authors' institution, banning authors from submitting work to ASN journals for varying lengths of time, and/or retraction of the published work.

Name: Sandhya Manohar
Manuscript ID: K360-2024-000797R1
Manuscript Title: Impact of an Interdisciplinary Chronic Kidney Disease Clinic on Disease Progression, Healthcare Use, and Social Determinants of Health
Date of Completion: January 23, 2025
Disclosure Updated Date: January 23, 2025

ASN American Society of Nephrology
JASN Journal of the American Society of Nephrology
KIDNEY360 Accessing Our World From Every Angle
CJASN Clinical Journal of the American Society of Nephrology

ASN Journal Disclosure Form

As per ASN journal policy, I have disclosed any financial relationships or commitments I have held in the past 36 months as included below. I have listed my Current Employer below to indicate there is a relationship requiring disclosure. If no relationship exists, my Current Employer is not listed.

M. Mateo reports the following:
Employer: Mayo Clinic

I understand that the information above will be published within the journal article, if accepted, and that failure to comply and/or to accurately and completely report the potential financial conflicts of interest could lead to the following: 1) Prior to publication, article rejection, or 2) Post-publication, sanctions ranging from, but not limited to, issuing a correction, reporting the inaccurate information to the authors' institution, banning authors from submitting work to ASN journals for varying lengths of time, and/or retraction of the published work.

Name: Maria B. Mateo
Manuscript ID: K360-2024-000797R1
Manuscript Title: Impact of an Interdisciplinary Chronic Kidney Disease Clinic on Disease Progression, Healthcare Use, and Social Determinants of Health
Date of Completion: December 13, 2024
Disclosure Updated Date: December 13, 2024

ASN American Society of Nephrology | JASN Journal of the American Society of Nephrology | KIDNEY360 Accessing Our World From Every Angle | CJASN Clinical Journal of the American Society of Nephrology

ASN Journal Disclosure Form

As per ASN journal policy, I have disclosed any financial relationships or commitments I have held in the past 36 months as included below. I have listed my Current Employer below to indicate there is a relationship requiring disclosure. If no relationship exists, my Current Employer is not listed.

A. Moran-Melendez has nothing to disclose.

I understand that the information above will be published within the journal article, if accepted, and that failure to comply and/or to accurately and completely report the potential financial conflicts of interest could lead to the following: 1) Prior to publication, article rejection, or 2) Post-publication, sanctions ranging from, but not limited to, issuing a correction, reporting the inaccurate information to the authors' institution, banning authors from submitting work to ASN journals for varying lengths of time, and/or retraction of the published work.

Name: Andrea C Moran-Melendez
Manuscript ID: K360-2024-000797R1
Manuscript Title: Impact of an Interdisciplinary Chronic Kidney Disease Clinic on Disease Progression, Healthcare Use, and Social Determinants of Health
Date of Completion: December 14, 2024
Disclosure Updated Date: December 14, 2024

ASN American Society of Nephrology | JASN Journal of the American Society of Nephrology | KIDNEY360 Accessing Our World From Every Angle | CJASN Clinical Journal of the American Society of Nephrology

ASN Journal Disclosure Form

As per ASN journal policy, I have disclosed any financial relationships or commitments I have held in the past 36 months as included below. I have listed my Current Employer below to indicate there is a relationship requiring disclosure. If no relationship exists, my Current Employer is not listed.

K. Rose reports the following:
Employer: Mayo Clinic

I understand that the information above will be published within the journal article, if accepted, and that failure to comply and/or to accurately and completely report the potential financial conflicts of interest could lead to the following: 1) Prior to publication, article rejection, or 2) Post-publication, sanctions ranging from, but not limited to, issuing a correction, reporting the inaccurate information to the authors' institution, banning authors from submitting work to ASN journals for varying lengths of time, and/or retraction of the published work.

Name: Katie Rose
Manuscript ID: K360-2024-000797R1
Manuscript Title: Impact of an Interdisciplinary Chronic Kidney Disease Clinic on Disease Progression, Healthcare Use, and Social Determinants of Health
Date of Completion: December 16, 2024
Disclosure Updated Date: December 16, 2024

ASN American Society of Nephrology
JASN Journal of the American Society of Nephrology
KIDNEY360 Accessing Our World From Every Angle
CJASN Clinical Journal of the American Society of Nephrology

ASN Journal Disclosure Form

As per ASN journal policy, I have disclosed any financial relationships or commitments I have held in the past 36 months as included below. I have listed my Current Employer below to indicate there is a relationship requiring disclosure. If no relationship exists, my Current Employer is not listed.

L. Salter has nothing to disclose.

I understand that the information above will be published within the journal article, if accepted, and that failure to comply and/or to accurately and completely report the potential financial conflicts of interest could lead to the following: 1) Prior to publication, article rejection, or 2) Post-publication, sanctions ranging from, but not limited to, issuing a correction, reporting the inaccurate information to the authors' institution, banning authors from submitting work to ASN journals for varying lengths of time, and/or retraction of the published work.

Name: Lucy Salter
Manuscript ID: K360-2024-000797R1
Manuscript Title: Impact of an Interdisciplinary Chronic Kidney Disease Clinic on Disease Progression, Healthcare Use, and Social Determinants of Health
Date of Completion: December 14, 2024
Disclosure Updated Date: December 14, 2024

ASN American Society of Nephrology | JASN Journal of the American Society of Nephrology | KIDNEY360 Accessing Our World From Every Angle | CJASN Clinical Journal of the American Society of Nephrology

ASN Journal Disclosure Form

As per ASN journal policy, I have disclosed any financial relationships or commitments I have held in the past 36 months as included below. I have listed my Current Employer below to indicate there is a relationship requiring disclosure. If no relationship exists, my Current Employer is not listed.

L. Vaughan reports the following:
Employer: Mayo Clinic

I understand that the information above will be published within the journal article, if accepted, and that failure to comply and/or to accurately and completely report the potential financial conflicts of interest could lead to the following: 1) Prior to publication, article rejection, or 2) Post-publication, sanctions ranging from, but not limited to, issuing a correction, reporting the inaccurate information to the authors' institution, banning authors from submitting work to ASN journals for varying lengths of time, and/or retraction of the published work.

Name: Lisa E. Vaughan
Manuscript ID: K360-2024-000797R1
Manuscript Title: Impact of an Interdisciplinary Chronic Kidney Disease Clinic on Disease Progression, Healthcare Use, and Social Determinants of Health
Date of Completion: January 23, 2025
Disclosure Updated Date: January 23, 2025

ASN American Society of Nephrology JASN Journal of the American Society of Nephrology KIDNEY360 Accessing Our World From Every Angle CJASN Clinical Journal of the American Society of Nephrology

ASN Journal Disclosure Form

As per ASN journal policy, I have disclosed any financial relationships or commitments I have held in the past 36 months as included below. I have listed my Current Employer below to indicate there is a relationship requiring disclosure. If no relationship exists, my Current Employer is not listed.

Z. Zoghby reports the following:
Employer: Mayo Clinic; Consultancy: Chronisense Medical, Ltd; BMJ Best Practice Expert Panel (Consutling); and Advisory or Leadership Role: EPIC corporation - Role (Unpaid): Chair of the Nephrology Steering Board Committee.

I understand that the information above will be published within the journal article, if accepted, and that failure to comply and/or to accurately and completely report the potential financial conflicts of interest could lead to the following: 1) Prior to publication, article rejection, or 2) Post-publication, sanctions ranging from, but not limited to, issuing a correction, reporting the inaccurate information to the authors' institution, banning authors from submitting work to ASN journals for varying lengths of time, and/or retraction of the published work.

Name: Ziad Zoghby
Manuscript ID: K360-2024-000797R1
Manuscript Title: Impact of an Interdisciplinary Chronic Kidney Disease Clinic on Disease Progression, Healthcare Use, and Social Determinants of Health
Date of Completion: February 4, 2025
Disclosure Updated Date: January 23, 2025
