# Supplementary material for: Effect of an Interdisciplinary CKD Clinic on Disease Progression, Health Care Use, and Social Determinants of Health
Source: Kidney360. 2025 Feb 18;6(6):937–46. doi: 10.34067/KID.0000000734 (PMC12233851; doi:10.34067/KID.0000000734)
Supplement: SUPPLEMENTARY MATERIAL [file kidney360-6-0937-s002.pdf]

Supplemental Materials
Table of Contents

Supplemental Table 1 1
Supplemental Material 1. Chronic Kidney Disease (CKD) Clinic Cadence 2
Supplemental Material 2. Protocol 6

Supplemental Table 1

Supplemental Table 1. Summary of patient encounter rates		
	**Pre-entry into CKD clinic**	**Post-entry into CKD clinic**
**N**	525	534
**Follow-up duration (days), median [IQR]**	181 [181, 182]	183 [181, 184]
**Patient Encounter Type**	**# of Visits, median [IQR]**	**# of Visits, median [IQR]**
General Hospital Admissions	0 [0, 1]	0 [0, 1]
ED visits	0 [0, 1]	0 [0, 0]
Nephrology visits (in-center or virtual)	1 [0, 2]	1 [1, 2]
Primary Care visits	1 [0, 2]	1 [0, 2]
CKD: Chronic Kidney Disease, ED: Emergency Department, IQR: Interquartile Range		

Supplemental Material 1. Chronic Kidney Disease (CKD) Clinic Cadence

**Chronic Kidney Disease Orders**<br>**Scope:** Mayo 19- Hypertension/Nephrology RNs<br>**Purpose:** To provide guidance to the RN when teeing-up orders for the provider as part of the pre-visit process.				
	Stage 3a	Stage 3b	Stage 4	Stage 5
**Appointments**				
Provider	Every 6 months	Every 3-6 months	Every 3-6 months	Every 4-6 weeks
**Labs**	Frequency			
CBC without differential	Every 6 months	Every 3-6 months	Every 3-6 months	Every 4-6 weeks
Renal function panel	Every 6 months	Every 3-6 months	Every 3-6 months	Every 4-6 weeks
Uric acid	Every 12 months	Every 12 months	Every 12 months	Every 6-12 months
PTH	Every 6 months	Every 3-6 months	Every 3-6 months	Every 3 months
Urinalysis	Every 6 months	Every 3-6 months	Every 3-6 months	
Urine albumin/creatinine ratio	Every 6 months	Every 3-6 months	Every 3-6 months	
**Anemia management labs**				
Iron and TIBC			If Hgb <10 every 3 months	If Hgb <10 every 3 months
Ferritin			If Hgb <10 every 3 months	If Hgb <10 every 3 months
Folic acid			If Hgb <10 at least once	If Hgb <10 at least once
Vitamin B12			If Hgb <10 at least once	If Hgb <10 at least once
**MBD Labs**				
25-hydroxy Vitamin D2 and D3	Once<br><br>Follow up Provider to enter	Once<br><br>Follow up Provider to enter	Provider to order	Provider to order
**Infectious screening**				
QuantiFERON TB Gold Plus			Provider to order	Within one year of starting dialysis.<br><br>Provider to order

HBc Total Ab, serum Hepatitis B Surface Antigen HBs antibody, serum			Provider to order	Every thirty days if no antibodies present, at discretion of provider.<br><br>Provider to order

HCV Ab scrn w/reflex to HCV PCR, serum			Provider to order	Provider to order
**Immunizations**				
Pneumococcal	Mayo Clinic has transitioned to 30-valent pneumococcal conjugate vaccine (PCV30) for all adults aged ≥ 65 years, and for adults aged 19-64 years with certain underlying *medical conditions or other risk factors who have not previously received a pneumococcal conjugate vaccine or whose previous vaccination history is unknown.<br>*Includes stage 3, 4, or 5 chronic renal failure<br><br>**Ask Mayo Expert-Risk Based Schedule**<br><br>Ages 19 to 64 with Stage 3, 4, or 5 chronic renal failure:<br>No prior pneumoccal vaccine:<br>• Single dose of PCV20<br>• Pneumococcal vaccination is then complete-no additional doses needed. If prior pneumococcal vaccine(s), refer to Ask Mayo Expert-Risk based schedule<br><br>Ages ≥ 65 with Stage 3, 4, or 5 chronic renal failure:<br>No prior pneumococcal vaccine:<br>• Single dose of PCV20<br>• Pneumococcal vaccination is then complete-no additional doses needed. If prior pneumococcal vaccine(s), refer to Ask Mayo Expert-Risk based schedule			
Influenza	Annual			
Hepatitis B	Strongly encourage to initiate catch up schedule if previously unvaccinated or if prior HBs Antibody Screen is negative.<br><br>If no prior HBs Antibody and patient is unaware of vaccination status, then consider ordering HBs Antibody for screening immunity status.<br><br>Order Recombivax HB DIALYSIS (3 doses 0,1,6 mons)			Confirm immune status with positive HBs Antibody or vaccination.<br><br>If not done, order to be tee-d up for provider. Order Recombivax HB DIALYSIS (3 doses 0,1,6 mons)
**Referrals**				
Education	Stage 3a- Keeping Your Kidneys Healthy (at least once)- HTN/Neph RN to tee-up order for provider		Kidney Care Advocates (KCA) will meet with patients with Stage 3b, 4, 5 and provide education on treatment options.<br><br>If transplant referral planned: Kidney Transplant referral/education - pre-education visit for Kidney or Kidney-pancreas (if diabetic)	

Transplant Referral	-	Kidney Transplant Referral order to be placed<br>-All patients if eGFR <20<br>-If eGFR <30 + diabetic<br>-if eGFR <30 + BMI >35<br>-if eGFR <30 + rapid decline in function

Access placement	--		Referral based on modality<br><br>(PD patients – one month before anticipated dialysis start)<br>(Not needed if preemptive<br>transplant is planned)
Dietician	Stage 3a and 3b: Once	Stage 4: Every 12 months	Stage 5: Every 6 months and as needed
Social Work	Stage 3a HTN/Neph RN or Provider: Place consult as needed based on clinical assessment.<br><br>*Include patient preference for visit (i.e., video, call, in-person)		Stage 3b, 4, 5: Providers and Kidney Care Advocates will place social work consult as needed based on clinical assessment.<br>*Include patient preference for visit (i.e., video, call, in-person)
Capacity Coaching			Per provider discretion

Guidance for Transplant Referral: Likely not a kidney transplant candidate if with any of the following (but all are relative contraindications and would use clinical judgement)

Age >80 with no living donors
Active Malignancy
Recurrent Chronic Infections
Immigrant/Refugee status on Emergency Medicaid only
Patient refusal for kidney transplant

Version History:
7/3/2024: Minor changes to social work, education, dietician sections based on stakeholder feedback. 6/10/2024: Multiple changes made during CKD meeting- Infectious Screening/Referrals sections 2/1/2024: Changed Appointment/Lab timeframes for Stage 3a from every 3-6 months to every 6 months. 12/19/2023: Updated Pneumococcal recommendations to include PCV20

Date of Last Review: 07/3/2024
Content Owner: Brigid Amberg, RN; Heather Stonelake-French, RN, CNS
Content Reviewers: Erin Dahlen, RN; Margaret (Peggy) d'Uscio, APRN, CNP, DNP

Supplemental Material 2. Protocol

Mayo Clinic Chart Review/Use of Existing Biospecimen Protocol Template

General Study Information
**Principal Investigator:** Kasey R. Boehmer, Sandhya M. Manohar; Ziad Zoghby<br>**Study Title:** Evaluating an Evidence Based Interdisciplinary CKD Clinic<br>**Protocol version number and date:** V3, 01/03/2022
**Overview**

Hypothesis (*Briefly state your hypothesis*): We hypothesize that the implementation of a novel Chronic Kidney Disease (CKD) clinic model will improve patient-important outcomes, including:

a. Increased number of patients choosing home dialysis over in-center hemodialysis
b. Increased number of patients receiving kidney transplant
c. Decreased number of dialysis starts in-hospital
d. Slower rate of progression from CKD to end-stage kidney disease
e. Decreased hospitalizations
f. Decreased patient illness intrusiveness from illness and treatment
g. Improved patient health outcomes for CKD

Aims of Analysis (*Briefly describe*):

The Division of Nephrology and Hypertension will be implementing a new evidence-based care model for Mayo Clinic patients receiving care for chronic kidney disease (CKD). The three core components of this model are to: 1) expand the Mayo Clinic home dialysis program through expansion of existing services and improved patient care coordination for current home dialysis recipients and 2) implement an interdisciplinary CKD clinic, that includes the addition of nurse navigators for CKD patients, definition and implementation of evidence-based care pathways, patient education plans, and shared decision making and care coordination support for patients as they transition to dialysis or palliative care; 3) care management oversight through EPIC registries.

Our aim is to examine whether the implementation of this program results in improved patient-important outcomes. To do so, we will examine several metrics collected by the practice, prior to and after implementation. We intend to examine changes from baseline to 3, 6, 12, 18, and 24-month outcomes.

Additionally, we will seek to compare outcomes of patient with CKD who are unplanned dialysis starts and receive care in our transitional care unit (TCU) compared to those that do not receive TCU care. The TCU is intensive multidisciplinary care provided in a 4-week time frame and intended to better prepare patients for their ESKD management strategy of choice (in-center dialysis, home dialysis, or palliative care). However, not all patients are able to or desire to receive TCU care.

Methods: *Briefly describe the research activities that will be conducted under this protocol.*
Include purpose, source, type and use of specimens and/or data.

We will use the following data from patient charts, administrative data, our CKD registry, dialysis registries, and our Tolvaptan registries:

a. CKD diagnosis date
b. Stage of CKD information over time, including progression to end-stage kidney disease (ESKD)
c. Dialysis modality selected and any changes over time
d. Transplant waitlisting and transplant completion dates
e. Healthcare utilization (e.g., number of visits, visit types)
f. Patient demographics (e.g., age, gender)
g. Patient social determinants of health (e.g., food insecurity)
h. Patient illness intrusiveness

i. Health outcomes for CKD (e.g. albumin)

The purpose of collecting this data will be to examine changes in patient-important outcomes associated with the implementation of the new CKD clinic model. For progression of illness outcomes, we will use time-to-event analyses. Count outcomes including healthcare utilization will be analyzed using negative binomial regression, and illness intrusiveness and continuous health outcome variables will be analyzed using OLS linear regression. All outcomes will be adjusted for patient demographics, social determinants, and primary clinic location. For patients that are transitioned to ESKD care, we will examine outcomes for those receiving TCU care vs non-TCU care. As such, for each patient receiving care in the TCU that is also eligible for chart review, we will find a similar matched patient eligible for chart review who transitioned to ESKD care but did not receive TCU care. We will match patients on age, sex, diabetes (yes/no), and heart failure (yes/no).

If the research will use data and/or specimens that have been collected under another IRB protocol, complete the following.

Enter one IRB number per line, add more lines as needed:

☐ Data ☐ Specimens ☐ Data & Specimens ___________________

☐ Data ☐ Specimens ☐ Data & Specimens ___________________

When appropriate, subjects must have provided consent allowing for future use of their data and/or specimens in the type of research described in this protocol.

Participant Information

Maximum accrual number: 2,000
Date Range for Specimens and/or Review of Medical Records: *6/1/2019 – 6/1/2024*

Note: The Date Range must include the period for collection of baseline data, as well as followup data, if applicable.

Study Population
Diagnosis/Disease of interest (*list all diagnoses that apply and check the appropriate subject population(s)*):

All patients with CKD followed in the CKD clinic.

Disease

☒ Male Adults (18+)
☒ Female Adults (18+)
☐ Male Children (<18)
☐ Female Children (<18)

Healthy/Control

☐ Male Adults (18+)
☐ Female Adults (18+)
☐ Male Children (<18)
☐ Female Children (<18)

Data Analysis

Indicate which of the following methods will be utilized for analysis of the data (*check all that apply and provide your descriptions*):

☐ Descriptive statistics will be utilized to broadly analyze the sample. No power calculation is needed.
Describe:

☒ Statistical modeling including multivariable modeling will be utilized and power calculations will be utilized when appropriate.
Describe: Assuming statistical test assumptions are met, for progression of illness outcomes, we will use time-to-event analyses. Count outcomes including healthcare utilization will be analyzed using negative binomial regression, and illness intrusiveness and continuous health outcome variables will be analyzed using OLS linear regression. All outcomes will be adjusted for patient demographics, social determinants, and primary clinic location.

☐ Formal modeling with unique data sets within the entire data group will be compared.
Describe:

Contact Health Sciences Research (HSR) Biomedical Statistics and Informatics (BSI) for help with your database or data analysis. https://pipeline.tcprod.mayo.edu/Pipeline/showrequest.html

Consent/HIPAA Waiver Criteria

Reminder: <u>All</u> regulatory waiver criteria must be met and justified for the IRB to consider a waiver.
Justification for waiver(s) must be provided in the IRBe application, as applicable.
The following regulatory information is provided as a reference for planning and development of your protocol.

Informed consent waiver criteria [45 CFR 46.116(d)]:
(1) The research involves no more than minimal risk to the subjects;

(2) The waiver or alteration will not adversely affect the rights and welfare of the subjects;

(3) The research could not practicably be carried out without the waiver or alteration; and

(4) Whenever appropriate, the subjects will be provided with additional pertinent information after participation.

HIPAA waiver criteria [45 CFR Part 164 - Security and Privacy Rule, Subpart E]:

(1) The use or disclosure of PHI involves no more than minimal risk to the privacy of individuals, based on the presence of at least the following elements:

An adequate plan to protect the identifiers from improper use and disclosure;

An adequate plan to destroy the identifiers at the earliest opportunity consistent with the conduct of the research, unless there is a health or research justification for retaining the identifiers or such retention is otherwise required by law; and

Adequate written assurances that the PHI will not be reused or disclosed to any other person or entity, except as required by law, for authorized oversight of the research study, or for other research for which the use or disclosure of PHI would be permitted by HIPAA.

(2) The research could not practicably be conducted without the waiver or alteration.
(3) The research could not practicably be conducted without access to and use of the PHI.
